# O-ring-induced transverse aortic constriction (OTAC) is a new simple method to develop cardiac hypertrophy and heart failure in mice

**DOI:** 10.1038/s41598-021-04096-9

**Published:** 2022-01-07

**Authors:** Yasuhisa Nakao, Jun Aono, Mika Hamaguchi, Kayo Takahashi, Tomohisa Sakaue, Katsuji Inoue, Shuntaro Ikeda, Osamu Yamaguchi

**Affiliations:** 1 Department of Cardiology, Pulmonology, Nephrology and Hypertension, Ehime University Graduate School of Medicine, Shitsukawa, Toon, Ehime 791-0295 Japan; 2 Department of Cardiovascular and Thoracic Surgery, Ehime University Graduate School of Medicine, Toon, Ehime Japan; 3 Department of Cell Growth and Tumor Regulation, Proteo-Science Center, Ehime University, Toon, Ehime Japan

**Keywords:** Cardiac hypertrophy, Heart failure

## Abstract

Suture-based transverse aortic constriction (TAC) in mice is one of the most frequently used experimental models for cardiac pressure overload-induced heart failure. However, the incidence of heart failure in the conventional TAC depends on the operator’s skill. To optimize and simplify this method, we proposed O-ring-induced transverse aortic constriction (OTAC) in mice. C57BL/6J mice were subjected to OTAC, in which an o-ring was applied to the transverse aorta (between the brachiocephalic artery and the left common carotid artery) and tied with a triple knot. We used different inner diameters of o-rings were 0.50 and 0.45 mm. Pressure overload by OTAC promoted left ventricular (LV) hypertrophy. OTAC also increased lung weight, indicating severe pulmonary congestion. Echocardiographic findings revealed that both OTAC groups developed LV hypertrophy within one week after the procedure and gradually reduced LV fractional shortening. In addition, significant elevations in gene expression related to heart failure, LV hypertrophy, and LV fibrosis were observed in the LV of OTAC mice. We demonstrated the OTAC method, which is a simple and effective cardiac pressure overload method in mice. This method will efficiently help us understand heart failure (HF) mechanisms with reduced LV ejection fraction (HFrEF) and cardiac hypertrophy.

## Introduction

Left ventricular (LV) hypertrophy occurs in response to chronic systemic hypertension and is an independent risk factor for HF^1^. Chronically elevated LV afterload is one of the leading causes of HF and is associated with high morbidity and mortality^2,3^. Therefore, research on cardiac hypertrophy is essential to prevent adverse cardiovascular events.

**Figure 1:**
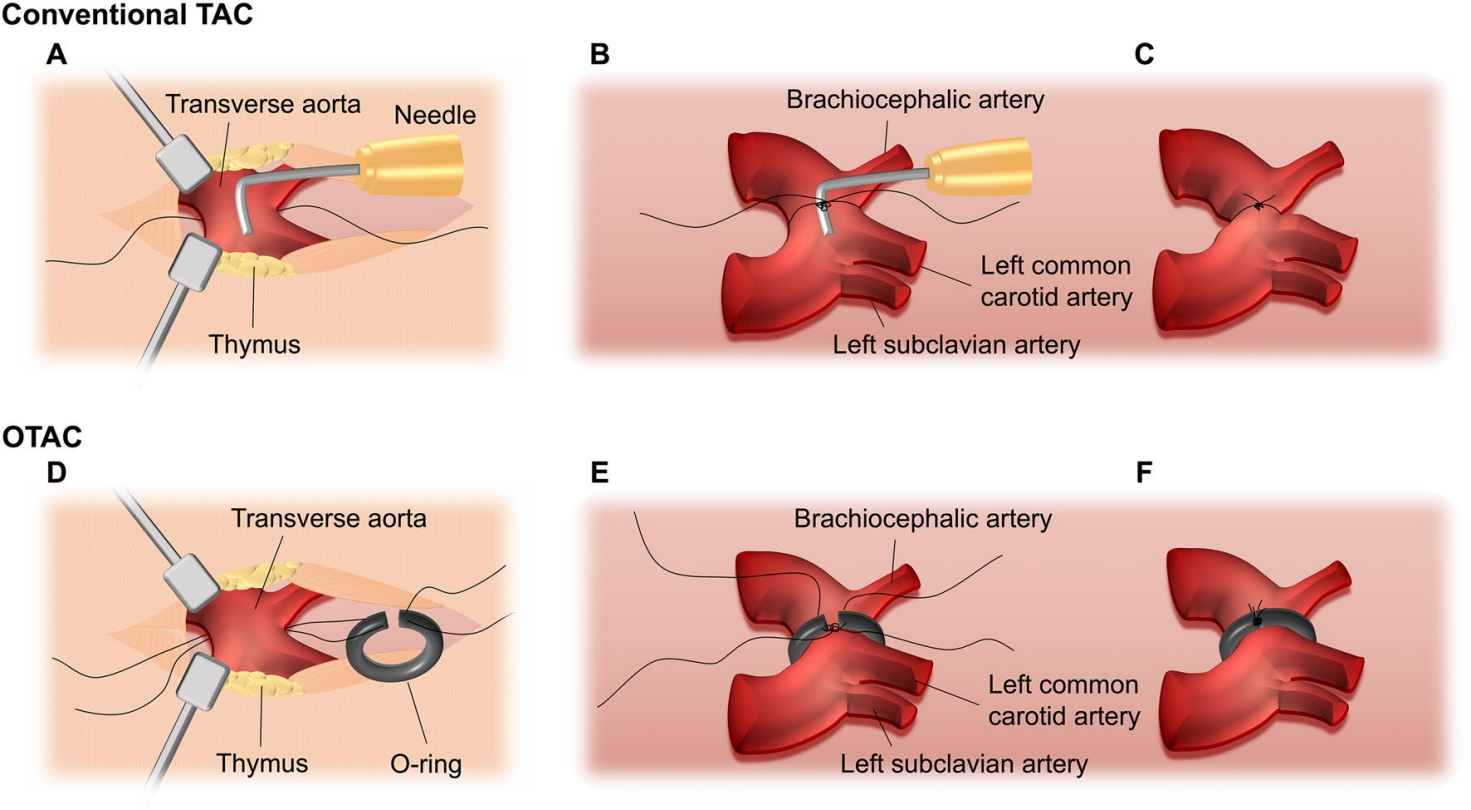
(**A–C**) Schematic of conventional transverse aortic constriction (TAC). (**A**) A small piece of a 6.0 silk suture is placed under the transverse aorta (between the brachiocephalic and left common carotid arteries). A blunt needle is placed parallel on the transverse aorta. (**B**) The suture is tied with triple knots against the needle. (**C**) The needle is promptly removed, and the excess suture is cut. (**D–F**) Schematic of O-ring-induced transverse aortic constriction (OTAC). (**D**) Two sutures penetrate the end of the o-ring (The details on the preparation of the o-ring are described in Supplemental Fig. 3). The o-ring is passed from the cranial side to the caudal side under the transverse aorta (between the brachiocephalic and left common carotid arteries). (**E**) The o-ring is placed around the transverse aorta. (**F**) Two sutures are tied with a triple knot, and the excess suture is cut. The details of the OTAC procedure are described in Supplemental Fig. 4.

Several animal models of cardiac hypertrophy, including isoproterenol^4–6^, angiotensin II infusion^7,8^, and aortic constriction^9–12^, have been developed to investigate the mechanisms underlying hypertrophy. Transverse aortic constriction (TAC) (Fig. 1A–C) in mice is widely used to induce pressure overload, resulting in concentric hypertrophy, interstitial fibrosis, and increased LV stiffness, eventually leading to systolic and diastolic heart failure^9–13^. This model is considered an excellent experimental tool for understanding the mechanisms of HF and cardiac hypertrophy over the past two decades. However, the conventional suture-based TAC is highly dependent on the operator due to the difficulty of the procedure, which can lead to variable degrees of aortic constriction. In addition, the range of mortality varies from 0 to 45%, depending on the reports^13–17^. Therefore, a simple model with hypertrophy and HF in mice is required.

Melleby et al. reported a new method, o-ring aortic banding (ORAB), which involves constriction of the ascending aorta of mice using an o-ring with fixed inner diameters^18^. ORAB has a highly reproducible pressure overload, resulting in highly reproducible cardiac remodeling. This method overcomes the drawbacks of the TAC procedure and is a novel standard method for the pressure overload-induced heart failure model in mice. However, this method required intubation for the operation, and the approach site was different from the traditional TAC procedure^18^. It is considered that the same approach as TAC is necessary for worldwide use of the procedure using an o-ring. Furthermore, the constriction of the ascending aorta leads to acute pressure overload in the LV chamber and provides rapid functional and morphological alteration in the left ventricle^19^. The TAC model showed a more gradual progression of hypertrophy compared to the ascending aortic constriction model.

Therefore, this study aimed to characterize the cardiac pressure overload that causes left-ventricular hypertrophy and HF in mice using the O-ring-induced transverse aortic constriction (OTAC) technique (Fig. 1D–F).

## Results

### Mortality rate of mice during and post OTAC procedure

**Figure 2:**
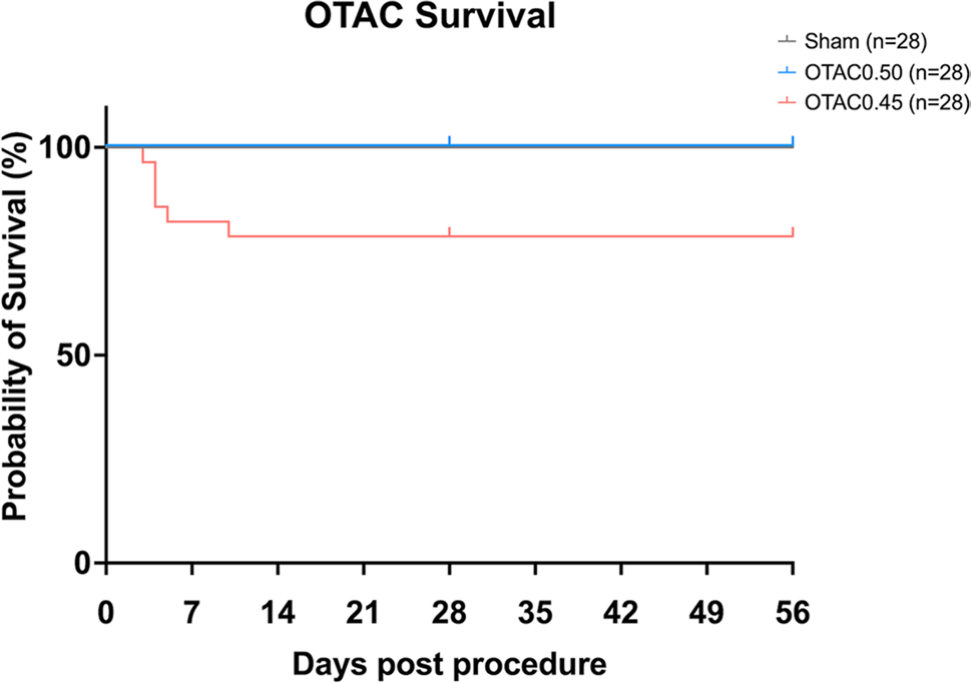
Kaplan–Meier survival curves for Sham and O-ring-induced transverse aortic constriction with an inner diameter of 0.50 mm (OTAC0.50) and 0.45 mm (OTAC0.45).

Baseline body weights were similar among all groups (Table 1). The average operation time (from thoracotomy to chest closure) for Sham, OTAC0.50, and OTAC0.45 was 8.2 ± 0.3, 17.3 ± 0.5, and 17.9 ± 0.6 min, respectively (Supplemental Fig. 1). The mortality rates for Sham, OTAC0.50, and OTAC0.45 mice during the procedure from anesthesia to chest closure were 0% (0/28), 9.7% (3/31), and 12.5% (4/32), respectively (Table 2). Thus, the total mortality rate during the OTAC procedure was 11.1% (7/63). The leading cause of death during the procedure was arterial bleeding from the aorta. The mortality rate in the early phase of the post-procedure period (from chest closure up to 48 h timepoint after the procedure) for Sham, OTAC0.50, and OTAC0.45 mice were all 0% (0/28) (Table 2). Hence, the seven OTAC mice that died during the procedure (n = 3, OTAC0.50 and n = 4, OTAC0.45) were not included in the survival analysis. The mortality rate in the late phase of the post-procedure period (after 48 h timepoint post-procedure) was 0% (0/28), 0% (0/28), and 17.9% (5/28) for Sham, OTAC0.50, and OTAC 0.45 mice, respectively (Table 2). Analysis of the Kaplan–Meier survival curve in the Sham and OTAC-operated groups revealed significantly higher mortality in the OTAC0.45 group than in the Sham and OTAC0.50 groups (Fig. 2). In this phase, the presumed cause of death in this phase was HF suspected by body weight loss with decreased LV systolic function and the dilatation of LV dimension without bleeding in the thoracic cavity by necropsies.

**Table 1:** Physical characteristics of mice subjected to O-TAC and sham procedures.

Variables	Weeks	Sham	OTAC0.50	OTAC 0.45
Body weight at baseline (g)	4	25.7 ± 0.25	25.7 ± 0.31	25.8 ± 0.40
	8	26.0 ± 0.33	26.1 ± 0.35	26.0 ± 0.39
Body weight at terminal sacrifice (g)	4	27.5 ± 1.04	27.2 ± 1.26	27.7 ± 1.16
	8	28.9 ± 0.74	29.7 ± 1.04	28.9 ± 3.59
Tibia length (mm)	4	16.8 ± 0.14	16.7 ± 0.16	16.7 ± 0.13
	8	17.5 ± 0.72	18.4 ± 0.27	16.9 ± 0.12
Heart weight/tibia length (mg/mm)	4	7.60 ± 0.61	10.2 ± 1.69*	10.5 ± 1.41*
	8	7.15 ± 0.69	12.6 ± 3.42*	14.5 ± 3.57*
LV weight/tibia length (mg/mm)	4	5.43 ± 0.40	7.75 ± 1.23*	8.18 ± 1.08*
	8	5.15 ± 0.55	9.20 ± 2.01*	10.8 ± 2.13*
Atrial weight/tibia length (mg/mm)	4	0.78 ± 0.24	0.92 ± 0.36	0.88 ± 0.30
	8	0.84 ± 0.22	1.42 ± 0.72*	1.42 ± 0.70
RV weight/tibia length (mg/mm)	4	1.26 ± 0.21	1.45 ± 0.33	1.35 ± 0.32
	8	1.22 ± 0.22	2.01 ± 1.86	2.13 ± 1.10*
Lung weight/tibia length (mg/mm)	4	8.36 ± 0.37	9.97 ± 4.88	10.0 ± 4.55
	8	8.51 ± 0.22	12.1 ± 7.96*	14.7 ± 8.95*

Data are expressed as the mean ± standard error of the mean. Physical characteristics in sham-operated and O-ring-induced transverse aortic constriction (OTAC) mice. O-rings had inner diameters of 0.50 mm (OTAC0.50) and 0.45 mm (OTAC0.45). n = 14 in the Sham group; n = 14 in O-TAC0.50 group; n = 9–13 in O-TAC0.45 group. Terminal sacrifice was performed in 4 weeks and 8 weeks after procedures. *LV* left ventricular, *RV* right ventricular. One-way analysis of variance (ANOVA) with Tukey’s post hoc tests were used (*P < 0.05 vs. sham).

**Table 2:** The mortality rate during and post procedure.

	Sham	OTAC0.50	OTAC0.45
During procedure (From anesthesia to chest closure)	0% (0/28)	9.7% (3/31)	12.5% (4/32)
Early phase post procedure (From chest closure up to 48 h timepoint after the procedure)	0% (0/28)	0% (0/28)	0% (0/28)
Late phase post procedure (After 48 h timepoint post-procedure)	0% (0/28)	0% (0/28)	17.9% (5/28)

The mice were performed sham procedure (Sham) or O-ring-induced transverse aortic constriction (OTAC). O-rings had inner diameters of 0.50 mm (OTAC0.50) and 0.45 mm (OTAC0.45).

### OTAC promotes myocardial hypertrophy and heart failure

**Figure 3:**
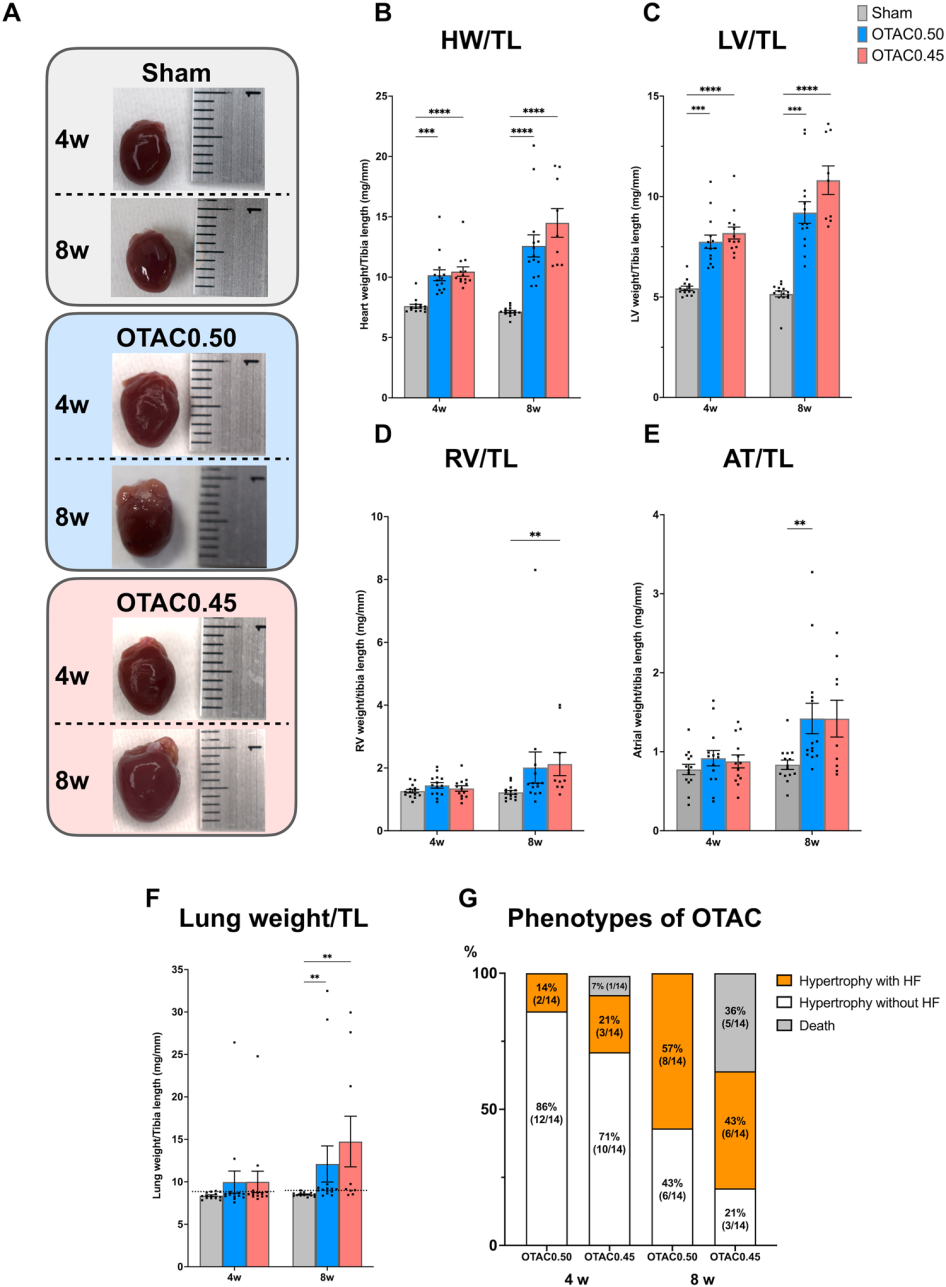
OTAC results in reproducible and graded myocardial hypertrophy. (**A**) Representative images showing cardiac morphology. (**B–E**) Bar graphs showing quantitative data for heart weight (HW)/tibia length (TL) (**B**), left ventricular (LV) weight/TL (**C**), right ventricular (RV) weight/TL (**D**) and atrial (AT) weight/TL (**E**). OTAC also results in reproducible lung congestion. (**F**) Lung weight normalized to TL. The dotted line indicates a maximum value for Sham mice. (**G**) Variability in cardiac phenotype in response to the duration of OTAC. These data were measured at terminal sacrifice at 4 weeks and 8 weeks after procedures. Data are expressed as the mean ± standard error of the mean. O-rings had inner diameters of 0.50 mm (OTAC0.50) and 0.45 mm (OTAC0.45). Comparison among groups was performed by one-way analysis of variance (ANOVA) with Tukey’s post hoc tests; n = 9–14. **P < 0.01; ***P < 0.001; ****P < 0.0001.

OTAC results in reproducible and graded myocardial hypertrophy. Representative images showing cardiac morphology (Fig. 3A). Pressure overload by OTAC promoted increased whole heart weight (Fig. 3B) and LV hypertrophy represented by increased LV weight/TL (Fig. 3C). At 4 weeks post-procedure, LV weight/TL was, on average 1.4-and 1.5-fold significantly higher in OTAC0.50 and OTAC0.45, respectively, than in Sham mice (Fig. 3C and Table 1). Moreover, at 8 weeks post-procedure, the whole heart and LV weight in OTAC-operated groups had significantly increased compared to the Sham group (Fig. 3B,C, and Table 1). LV weight/TL was, on average, 1.8-and 2.1-fold significantly higher in OTAC0.50 and OTAC0.45, respectively, than in Sham mice (Fig. 3C and Table 1). The highest increase in LV weight/TL was observed for OTAC0.45. Thus, OTAC resulted in a reproducible and graded hypertrophic response. In addition, the right ventricle (RV)/TL weight of OTAC groups was significantly higher than in Sham at 8 weeks post-procedure (Fig. 3D and Table 1). The atria (AT)/TL weight of OTAC0.50 was significantly higher in Sham at 8 weeks post-procedure.

Pressure overload by OTAC also promoted increased lung weight, indicating severe pulmonary congestion, as previously reported^13,20^. At 4 weeks post-procedure, the lung weight/TL ratio was on average 1.2-fold higher in both OTAC0.50 and OTAC0.45, respectively, than in Sham mice (Fig. 3F and Table 1). At 8 weeks post-procedure, the lung weight/TL ratio significantly increased by 1.4-and 1.7-fold in OTAC0.50 and OTAC0.45, respectively, compared to Sham (Fig. 3F and Table 1). The highest increase in lung weight/TL was observed for OTAC0.45. Further analysis revealed that at 4 weeks post-procedure, 14% (2/14) of OTAC0.50 and 21% (3/14) of OTAC0.45 mice developed a lung weight/TL ratio above the maximum value for Sham mice, and at 8 weeks post-procedure, 57% (8/14) of OTAC0.50 and 43% (6/14) of OTAC0.45 mice developed a lung weight/TL ratio above the maximum value for Sham mice (Fig. 3G and Table 1).

### Echocardiographic findings of OTAC-induced cardiac hypertrophy and reduced cardiac function

**Figure 4:**
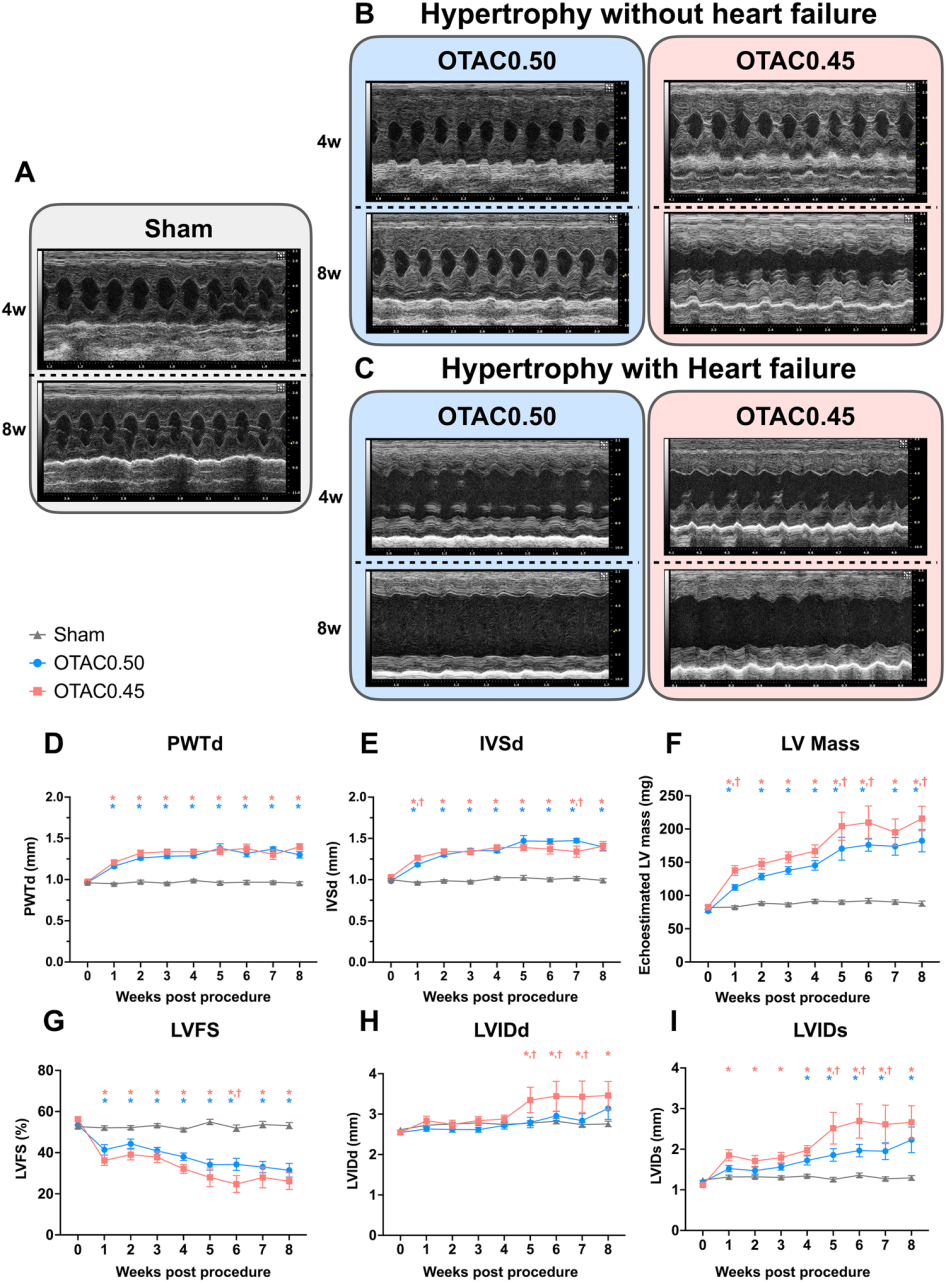
Echocardiographic analyses before and after OTAC and Sham. (**A–C**) Representative echocardiographic M-mode of mice 4 weeks and 8 weeks after the procedure; Sham (**A**), hypertrophy by OTAC (**B**), and heart failure with hypertrophy by OTAC (**C**). The x-axis represents the time (in ms), and the y-axis represents the distance (in mm) from the transducer. (**D–I**) Echocardiographic measurements of left ventricular posterior wall thickness in diastole: PWTd (**D**), interventricular septum in diastole: IVSd (**E)**, LV mass (**F**), LV fractional shortening: FS (**G**), LV internal dimension in end-diastolic dimension: LVIDd (**H)** and end-systolic dimension: LVIDs (**I**) were measured at 8 weeks post-procedure. Repeated measurement among groups was performed by two-way ANOVA with Tukey’s post hoc test; n = 9–14. *P < 0.05 versus Sham; ^†^*P* < 0.05 versus OTAC0.50.

In response to pressure overload, echocardiography showed that PWTd and IVSd had significantly increased in both OTAC groups than the Sham group at 1–8 weeks post-procedure (Fig. 4A–E and Table 3). The LV mass also increased during hypertrophy (Fig. 4F). To determine whether the increased mortality and incidence of HF by OTAC, which was related to a more severe cardiac dysfunction, we also assessed LV systolic function by echocardiography post-procedure. Echocardiographic analysis revealed that LVFS was reduced early in both OTAC groups and continued to decline (Fig. 4G and Table 3). LVFS in both OTAC groups was significantly reduced compared to the Sham group at 1–8 weeks. LVIDd was significantly larger in OTAC0.45 at 4–8 weeks, and LVIDs were significantly larger in OTAC0.50 at 4–8 weeks and in OTAC0.45 at 1–8 weeks than in the Sham group (Fig. 4H, I and Table 3). Representative cardiac hypertrophy and HFrEF cases are shown in Fig. 4A–C.

**Table 3:** Echocardiographic characteristics of mice subjected to OTAC and sham procedures.

	Operation	n	IVSd (mm)	PWTd (mm)	LVIDd (mm)	LVIDs (mm)	FS (%)	HR (bpm)
BL	Sham	28	1.00 ± 0.07	0.96 ± 0.06	2.61 ± 0.24	1.24 ± 0.17	52.7 ± 4.2	716 ± 30.3
	OTAC0.50	28	0.98 ± 0.08	0.97 ± 0.09	2.54 ± 0.29	1.19 ± 0.19	53.4 ± 4.6	718 ± 35.3
	OTAC0.45	28	1.03 ± 0.07	0.98 ± 0.08	2.55 ± 0.27	1.12 ± 0.20	56.3 ± 5.2	721 ± 48.8
1w	Sham	28	0.96 ± 0.07	0.95 ± 0.06	2.73 ± 0.28	1.31 ± 0.24	52.1 ± 5.2	734 ± 39.1
	OTAC0.50	28	1.18 ± 0.09*	1.16 ± 0.11*	2.63 ± 0.31	1.52 ± 0.41	41.4 ± 13.4*	735 ± 38.1
	OTAC0.45	23	1.27 ± 0.13*^†^	1.21 ± 0.11*	2.84 ± 0.53	1.84 ± 0.66*	36.2 ± 11.9*	698 ± 75.3*^†^
2w	Sham	28	0.99 ± 0.07	0.97 ± 0.106	2.76 ± 0.21	1.32 ± 0.19	52.2 ± 5.2	750 ± 38.8
	OTAC0.50	28	1.30 ± 0.12*	1.26 ± 0.11*	2.62 ± 0.34	1.47 ± 0.46	44.3 ± 12.1*	756 ± 40.5
	OTAC0.45	22	1.34 ± 0.14*	1.32 ± 0.17*	2.75 ± 0.49	1.71 ± 0.63*	39.0 ± 11.9*	728 ± 53.2
3w	Sham	28	0.98 ± 0.07	0.96 ± 0.07	2.78 ± 0.27	1.30 ± 0.21	53.3 ± 4.7	751 ± 41.6
	OTAC0.50	28	1.35 ± 0.14*	1.28 ± 0.14*	2.61 ± 0.32	1.56 ± 0.40*	40.8 ± 9.26*	758 ± 33.3
	OTAC0.45	22	1.34 ± 0.17*	1.35 ± 0.19*	2.84 ± 0.47	1.79 ± 0.60*	37.8 ± 12.2*	739 ± 50.4
4w	Sham	28	1.02 ± 0.07	0.99 ± 0.07	2.74 ± 0.30	1.34 ± 0.21	51.2 ± 4.4	764 ± 43.3
	OTAC0.50	28	1.35 ± 0.13*	1.29 ± 0.11*	2.72 ± 0.48	1.72 ± 0.60*	38.0 ± 9.6*	746 ± 40.4
	OTAC0.45	22	1.39 ± 0.12*	1.34 ± 0.13*	2.88 ± 0.47	1.97 ± 0.54*	32.1 ± 10.1*	737 ± 51.6
5w	Sham	14	1.02 ± 0.10	0.96 ± 0.07	2.78 ± 0.22	1.25 ± 0.19	54.9 ± 4.9	761 ± 29.2
	OTAC0.50	14	1.47 ± 0.24*	1.38 ± 0.20*	2.79 ± 0.48	1.86 ± 0.57*	34.1 ± 9.9*	758 ± 28.4
	OTAC0.45	9	1.39 ± 0.11*	1.35 ± 0.14*	3.35 ± 0.94*^†^	2.52 ± 1.17*^†^	28.0 ± 13.7*	712 ± 86.1
6w	Sham	14	1.00 ± 0.07	0.97 ± 0.08	2.83 ± 0.25	1.36 ± 0.20	51.9 ± 5.9	762 ± 33.8
	OTAC0.50	14	1.46 ± 0.11*	1.32 ± 0.16*	2.96 ± 0.41	1.97 ± 0.58*	34.3 ± 11.0*	747 ± 29.3
	OTAC0.45	9	1.37 ± 0.17*	1.38 ± 0.15*	3.44 ± 1.11*^†^	2.69 ± 1.26*^†^	24.7 ± 12.3*	676 ± 102*^†^
7w	Sham	14	1.02 ± 0.08	0.97 ± 0.06	2.74 ± 0.18	1.27 ± 0.18	53.6 ± 5.9	783 ± 33.9
	OTAC0.50	14	1.47 ± 0.10*	1.37 ± 0.10*	2.84 ± 0.64	1.95 ± 0.77*	33.0 ± 10.3*	752 ± 44.8
	OTAC0.45	9	1.34 ± 0.20*	1.30 ± 0.17*	3.41 ± 1.18*^†^	2.64 ± 1.40*^†^	26.4 ± 13.2*	672 ± 95.9*^†^
8w	Sham	14	0.99 ± 0.08	0.96 ± 0.07	2.76 ± 0.25	1.29 ± 0.20	53.2 ± 5.5	767 ± 21.6
	OTAC0.50	14	1.40 ± 0.16*	1.30 ± 0.14*	3.14 ± 1.02	2.23 ± 1.18*	31.5 ± 12.5*	737 ± 30.0
	OTAC0.45	9	1.39 ± 0.19*	1.38 ± 0.15*	3.47 ± 1.03*	2.69 ± 1.27*	25.6 ± 13.0*	672 ± 120*^†^

Data are expressed as the mean ± standard error of the mean. M-mode echocardiographic measurements of cardiac dimensions and function in sham-operated and O-ring-induced transverse aortic constriction (OTAC) mice at baseline (BL), 1, 2, 3, 4, 5, 6, 7 and 8 weeks after surgery. O-rings had inner diameters of 0.50 mm and 0.45 mm. Structural dimensions were measured in diastole (d) and systolic (s). IVS; interventricular septum, PWT; left ventricular posterior wall thickness, LVID; left ventricular inner diameter, FS; fractional shortening, HR; heart rate, BW; body weight. Repeated measures two-way ANOVA with Tukey’s post-hoc test was used (*P < 0.05 vs. Sham, ^†^*P* < 0.05 vs. OTAC 0.50).

### Cardiac hypertrophy in cardiac histology

**Figure 5:**
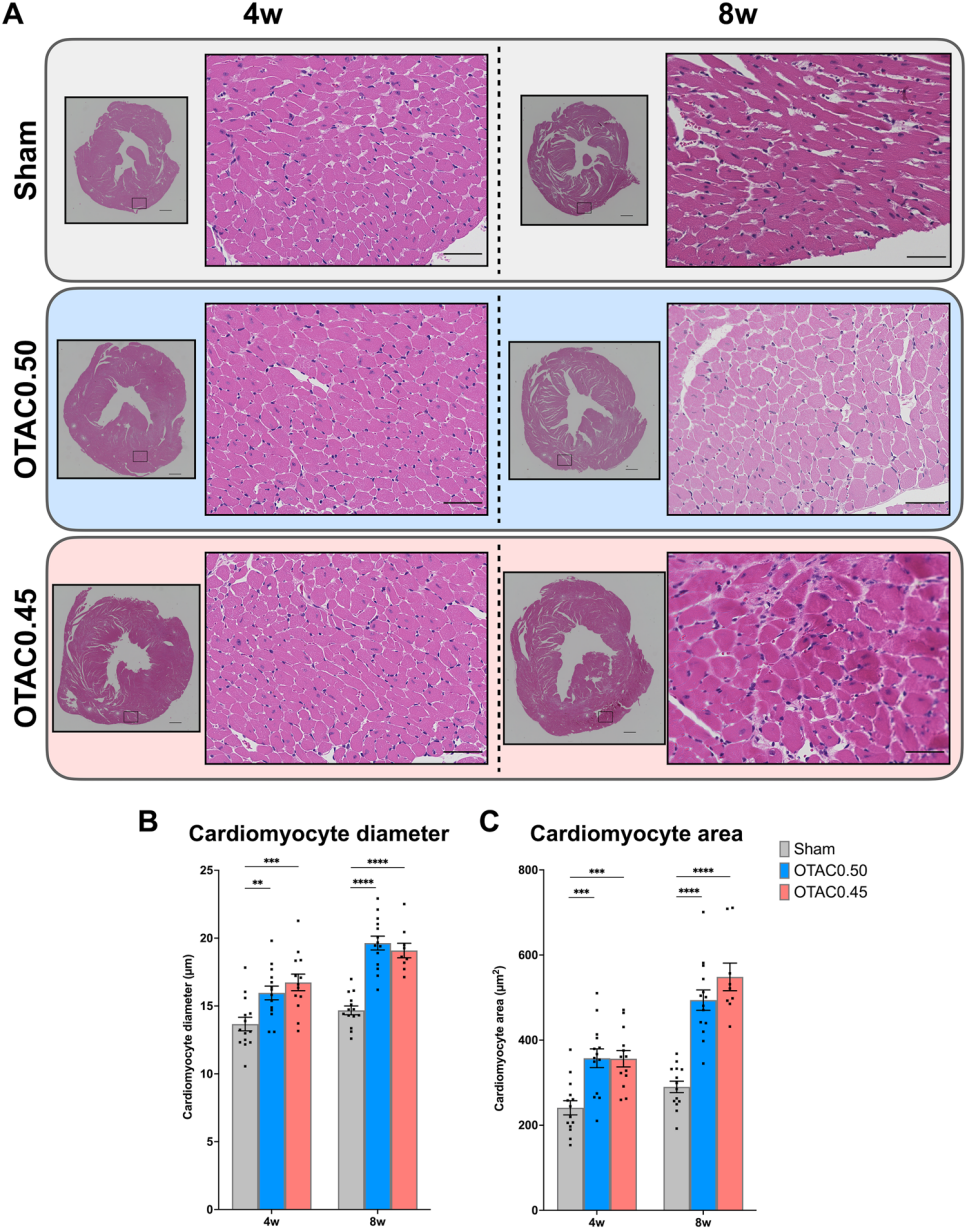
Cardiac histology at 4 and 8 weeks post-procedure. (**A**) Representative images of short-axis cardiac sections with hematoxylin and eosin staining in Sham and OTAC. Left: 4 × magnification of left ventricular at mid-ventricular sections. Scale = 500 µm. Right: 40 × magnification of a representative area. Scale = 100 µm. (**B**) Quantification of cardiomyocyte diameter of the short axis. (**C**) Quantification of cardiomyocyte area. Comparison among groups was performed by one-way ANOVA with Tukey’s post hoc tests; n = 9–14. **P < 0.01; ***P < 0.001; ****P < 0.0001.

We measured the cross-sectional area of cardiac myocytes at the terminal harvest (4 and 8 weeks after the procedure). Figure 5A shows representative images of HE staining in cardiac sections of OTAC and Sham mice at 4 and 8 weeks after the procedure. The length of the short axis in the cardiomyocytes was longer in both OTAC groups than in the Sham group [4 weeks: 13.7 ± 0.5 µm (Sham), 16.1 ± 0.5 µm (OTAC0.50), and 16.7 ± 0.6 µm (OTAC0.45), 8 weeks: 14.7 ± 0.3 µm (Sham), 19.6 ± 0.5 µm (OTAC0.50), and 19.1 ± 0.5 µm (OTAC0.45)] (Fig. 5B and Table 4). Similarly, the cardiac myocyte area in both OTAC groups was larger than that in the Sham group [4 weeks: 241 ± 16 µm^2^ (Sham), 358 ± 22 µm^2^ (OTAC0.50), and 356 ± 19 µm^2^ (OTAC0.45), 8 weeks: 290 ± 13 µm^2^ (Sham), 494 ± 24 µm^2^ (OTAC0.50), 549 ± 33 µm^2^ (OTAC 0.45)] (Fig. 5C and Table 4).

**Table 4:** Average measurements of myocardial tissue structure for each group in mice.

	Operation	n	Myocyte diameter (µm)	Myocyte area (µm^2^)
4w	Sham	14	13.7 ± 0.50	241 ± 16.7
	OTAC0.50	14	16.1 ± 0.46*	358 ± 22.0*
	OTAC0.45	13	16.7 ± 0.61*	356 ± 19.2*
8w	Sham	14	14.7 ± 0.32	290 ± 13.5
	OTAC0.50	14	19.6 ± 0.51*	494 ± 23.8*
	OTAC0.45	9	19.1 ± 0.54*	548 ± 32.6*

Data are expressed as the mean ± standard error of the mean. Myocyte cross-sectional area and diameter of short axis were measured in sham mice or O-ring induced transverse aortic constriction (OTAC) mice at terminal sacrifice. Terminal sacrifice was performed in 4 weeks and 8 weeks after procedures. O-rings had inner diameters of 0.50 mm (OTAC0.50) and 0.45 mm (OTAC0.45). n = 14 in the Sham group; n = 14 in O-TAC0.50 group; n = 9–13 in O-TAC0.45 group. Comparison among groups were performed by one-way analysis of variance (ANOVA) with Tukey’s post hoc tests (*P < 0.05 vs. Sham).

### OTAC promotes cardiac fibrosis

**Figure 6:**
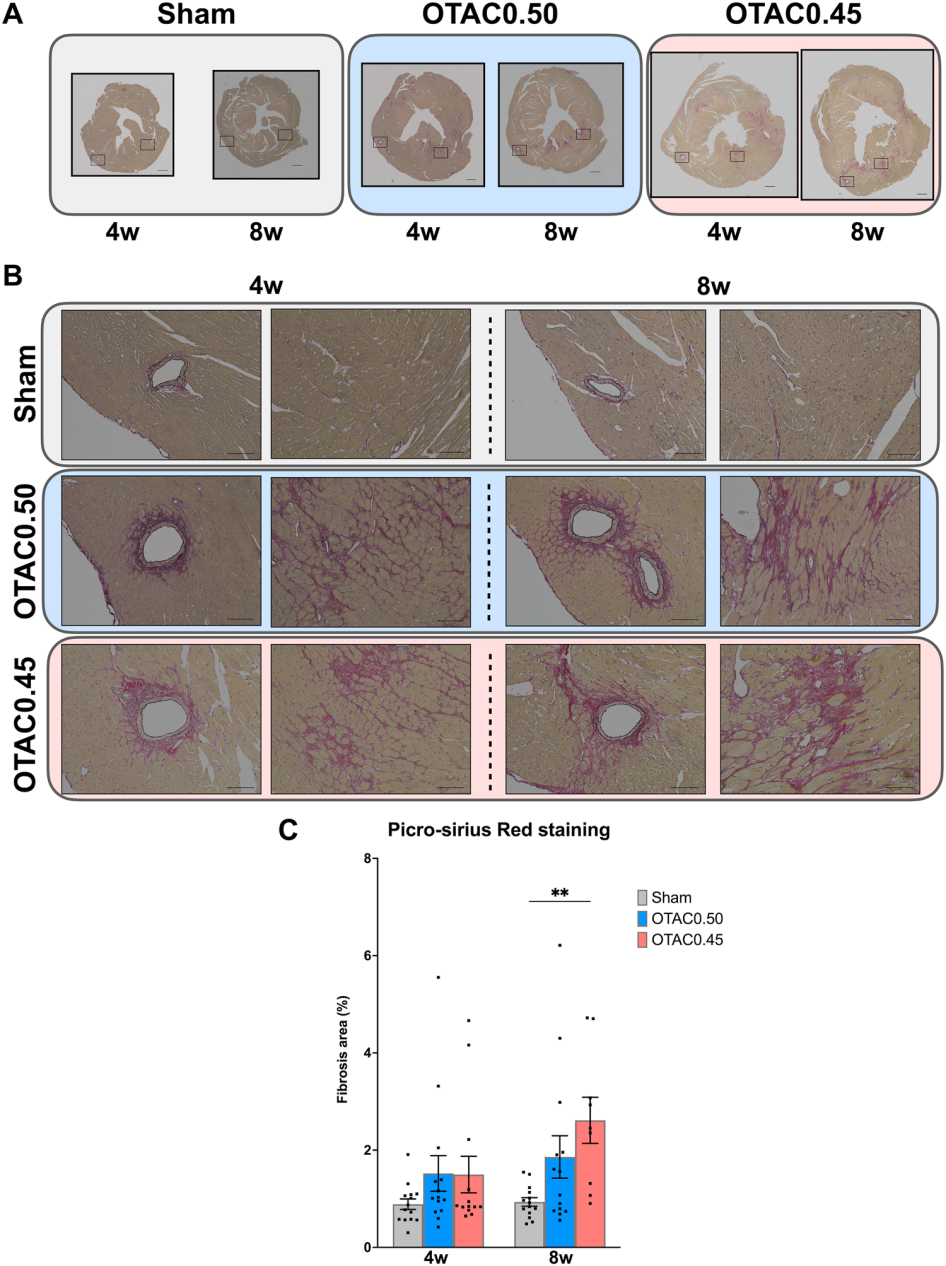
Histological analysis of left ventricular tissue showing graded cardiac fibrosis. Representative images of short-axis cardiac sections with Sirius-red staining in Sham and OTAC 4 and 8 weeks post procedures (**A, B**). (**A**) ×4 magnification of left ventricular at mid-ventricular sections. Scale = 500 µm. (**B**) Left: ×40 magnification of a representative area of perivascular fibrosis. Scale = 100 µm. (**B**) Right: ×40 magnification of a representative area of interstitial fibrosis. Scale = 100 µm. (**C**) Quantification of left ventricular fibrosis (% of the area of mid-ventricular sections) 4 and 8 weeks post procedures. Comparison among groups was performed by one-way ANOVA with Tukey’s post hoc tests; n = 9–14. **P < 0.01.

LV fibrosis was assessed in picrosirius red-stained sections at 4 and 8 weeks after the procedure. Representative LV fibrosis is shown in Fig. 6A,B. There were significantly increased in both OTAC groups compared to the Sham group [4 weeks: 0.9 ± 0.1% (Sham), 1.5 ± 0.4% (OTAC0.50) and 1.5 ± 0.4% (OTAC0.45), 8 weeks: 0.9 ± 0.3% (Sham), 1.9 ± 0.4% (OTAC0.50) and 2.6 ± 0.5% (OTAC0.45)] (Fig. 6C).

### Signature molecules of cardiac remodeling, failure, and fibrosis

**Figure 7:**
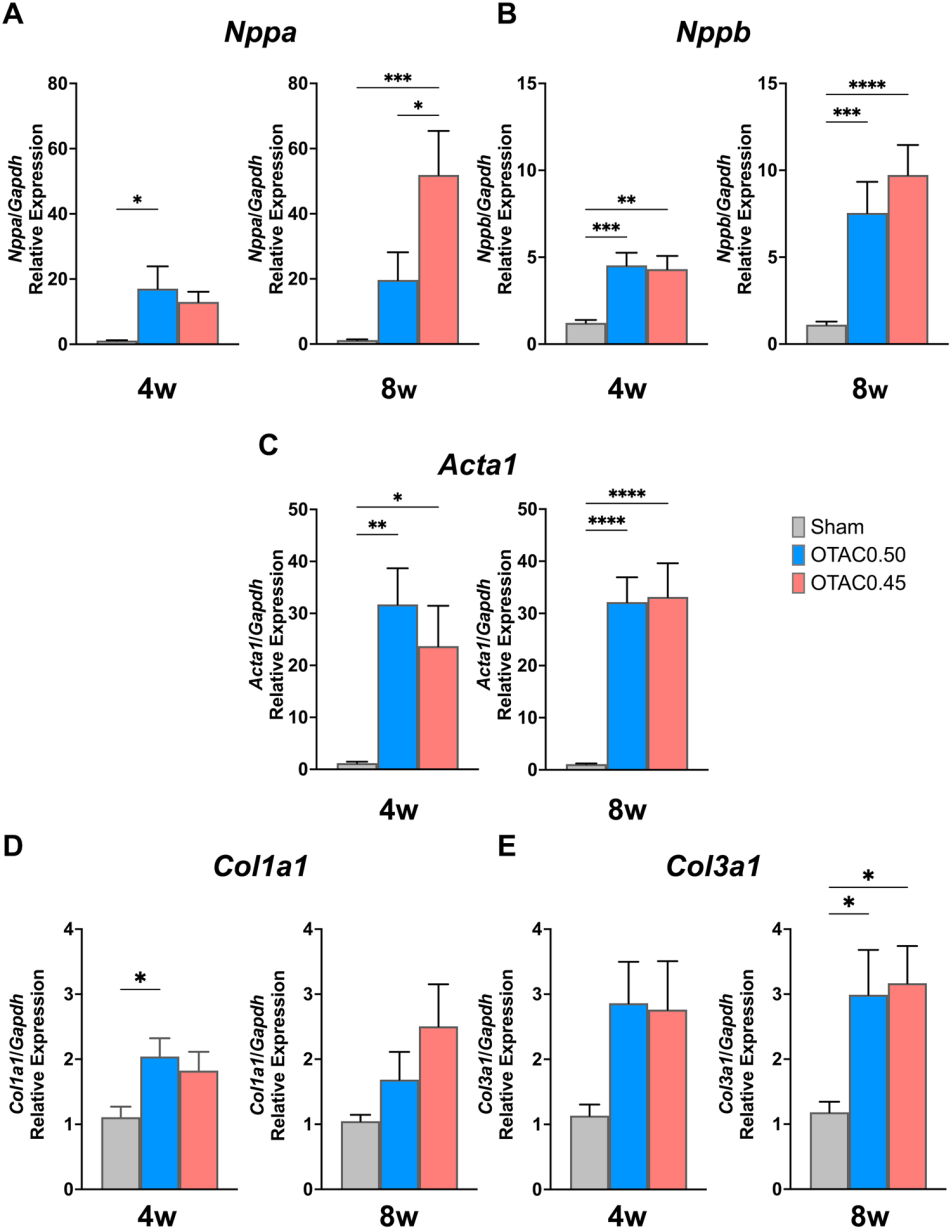
Data are expressed as the mean ± standard error of the mean. O-rings had inner diameters of 0.50 mm (OTAC0.50) and 0.45 mm (OTAC0.45). Terminal sacrifice was performed in 4 weeks and 8 weeks after procedures. Relative left ventricular (LV) mRNA levels of *Nppa* (**A**), *Nppb* (**B**), *Acta1* (**C**), *Col1a1* (**D**), and *Col3a1* (**E**) among groups at 4- and 8-weeks post procedure. Comparison among groups was performed by one-way analysis of variance (ANOVA) with Tukey’s post hoc tests; n = 9–14. *P < 0.05; **P < 0.01; ***P < 0.001; ****P < 0.0001.

In mice that survived 4 and 8 weeks after the procedure, we performed real-time quantitative PCR (qPCR) to determine the gene expression levels of the markers of HF, hypertrophy, and fibrosis in the LV of OTAC-induced HF model mice. The mRNA expression levels of natriuretic atrial peptide (*Nppa*), natriuretic brain peptide (*Nppb*), and actin, alpha 1, skeletal muscle (*Acta1)* were significantly higher in both OTAC groups than in the Sham group (Fig. 7A–C).

We also assessed the gene expression of fibrosis-related molecules in the myocardial tissue. The mRNA levels of collagen type Iα1 (*Col1a1*) had significantly increased in OTAC0.50 at 4 weeks and OTAC0.45 at 8 week, and collagen type IIIα1 (*Col3a1*) were significantly increased in both OTAC groups compared to the Sham group at 4 and 8 weeks (Fig. 7D,E). These results are consistent with the histological findings.

## Discussion

This study demonstrated an innovative method to induce cardiac pressure overload using the OTAC technique in mice. This method can be easily performed without intubation and is highly reproducible for simulating cardiac hypertrophy, fibrosis, and HFrEF (Tables 1 and 3). In addition, the lower inner diameters of the o-ring can induce an increasing degree of LV hypertrophy, LV dysfunction, and lung congestion. Our method can also be applied to cardiac hypertrophy models with or without HF by simply selecting an appropriate-sized o-ring. This feature is similar to the selection of the needle size in a conventional TAC procedure^13^.

Various surgical approaches have been developed to mimic patients with hypertensive heart disease to study heart failure with reduced ejection fraction induced by chronic pressure overload of the left ventricle. Rockman et al. first reported the TAC method^9^, the most widely used to study the LV pressure overload that induces HFrEF^21,22^. TAC causes an increase in afterload due to stenosis of the transverse aorta, resulting in concentric hypertrophy, interstitial and perivascular fibrosis, and HFrEF^9,12,13,23,24^. However, the TAC procedure has certain drawbacks. As it is highly operator-dependent, it has inter-operator variability and is technically demanding. This drawback might lead to variable degrees of aortic constriction, resulting in heterogeneous phenotypes. The mortality rate of mice undergoing TAC is difficult to estimate because the exact cause of the death is often not declared, and only successfully performed TAC procedures have been mentioned. Surgical variables such as mechanical or spontaneous ventilation^15^, type of approach (thoracotomy^9^ vs. mini-sternotomy^15^), site of the constriction, severity of the stenosis produced (cannula size^13^), and operator experience could affect the mortality in the conventional mice TAC model. Therefore, the mortality range in the TAC mouse model varies remarkably in several studies^15,16^.

Recently, two modified aortic constriction methods have been reported. One is the double-loop-clip technique^25^. In this method, the transverse aorta diameter is measured using echocardiography before the procedure to calculate the inter-knot span of the suture, which can decrease variability. This new procedure results in a far more accurate, reproducible stenosis that decreases mouse mortality and increases the homogeneity of structural and molecular features after aortic constriction^25^. The other method is the ORAB method^18^. This method involves constriction of the ascending aorta of mice using an o-ring with fixed inner diameters. A fixed inner diameter can easily achieve ascending aorta constriction for hypertrophy and HF. These methods are more reproducible than conventional TAC and may be used in place of TAC in the future, further advancing HF research.

In addition to the two modified aortic constriction methods, our proposed OTAC method has various advantages. First, a pressure overload can be applied by tying the thread on the o-ring [Fig. 1D–F and Supplemental Fig. 4 (10, 11)]. In the conventional TAC method, there is a possibility that the thread may loosen when the needle is removed from the thread; this is one of the causes of phenotypic variation and is improved by OTAC, wherein there is no concern regarding the thread loosening. The other possible cause was intraluminal suture migration. This phenomenon, resulting from tying with single-loop banding, appears in up to 30% of the animals after the conventional TAC procedure^26,27^. The o-ring, which is made of non-slip rubber and has a width greater than that of the thread, helps overcome this drawback. OTAC can develop concentric hypertrophy, interstitial and perivascular fibrosis, and has a high incidence of HF compared to conventional methods. (Fig. 3, Tables 1 and 3). In the comparison between OTAC (the surgeon with about 100 case experiences of OTAC and 20 case experiences of TAC) and TAC using the needle of 26 gauge (26G) (the surgeon with about 350 case experiences of TAC) in our laboratory, IVSd and PWTd were higher, and LVFS was lower in OTAC mice than 26G TAC mice (Supplemental Fig. 2). The number of cases experienced by the surgeons was relatively small, and they were considered to be beginners. Mice of 26G TAC were performed for the same weeks, body weight, and C57BL/6 J mouse as those of OTAC. This result shows that OTAC is a useful method for beginners to create the HFrEF model easily. Moreover, the features of HFrEF in our model are more represented than those in previously reported TAC mouse models^14–16^. OTAC can be easily diverted from the conventional TAC, and it may be useful even in laboratories with expertise in the conventional TAC mouse model. Second, OTAC can perform with mini-sternotomy and does not require intubation. The fact that OTAC does not require intubation of the mouse is an important advantage over ORAB. In addition, perioperative blood loss is minimal, and there was no risk of complications associated with intubation during OTAC. Anesthesia during OTAC involves intraperitoneal anesthesia. Therefore, the time needed for intubation can be reduced. In addition, the cost of the o-ring is low. Third, periprocedural mortality is likely comparable to that of conventional TAC. Periprocedural mortality is an essential point from an animal welfare perspective. According to past TAC reports, the mortality rate is approximately 0–45%^13–17^, which is considered acceptable from the point of our mortality rate of 11% (Table 4). OTAC is placed on the transverse aorta, which may slowly progress toward hypertrophy and HF compared to the constriction of the ascending aorta with o-rings. Although we could not directly compare OTAC and ORAB in this study, hemodynamics and phenotypes depending on the location of aortic constriction have been previously reported^28^. The constriction of the ascending aorta leads to the immediate onset of pressure overload and trauma to the vascular wall. Additionally, aortic occlusion produces LV overdistention and acute myocardial damage that may kill the animal or exhibit myocardial artifacts at termination studies. This occlusion is particularly relevant for ascending aortic constriction techniques^17,29,30^, where catastrophic consequences quickly follow complete aortic occlusion. OTAC for the transverse aorta could prevent these traumas, because in this procedure, the presumed cause of death post-procedure has been suspected to be HF without bleeding in the thoracic cavity by necropsies. Moreover, unlike the TAC procedure, it is possible to reduce the mortality rate during the surgery such that the OTAC could avoid complete occlusion of the aorta^29,31^.

This OTAC method may also have better reproducibility of HF occurrence if the following features are improved. OTAC used the same inner diameter of o-rings regardless of the body weight and aortic size of the mice. O-rings form similar aortic lumen areas in the mice despite different aortic diameters. It has been reported that the double loop-clip technique causes a similar percent reduction of aortic flow area in dissimilar animals using aortic diameter measured by echocardiographic techniques before the TAC procedure^25^. Similarly, we believe that measuring the aortic diameter of mice by echocardiographic techniques before OTAC and using an appropriate size of the o-ring may lead to high reproducibility of left ventricular hypertrophy and HF in mice.

The hypertrophic response to pressure overload and progression to HF depends on the genetic background of the mice. Lorena et al. reported that the C57BL/6J substrain appears to be a model of sustained cardiac hypertrophy, and C57BL/6NCrl and C57BL/6NTac substrains are more suitable for HF^20^. Therefore, according to this report, OTAC may be more reproducible in C57BL/6NCrl and C57BL/6NTac substrain mice for HF studies.

The severity of the constriction is usually assessed by measuring pulsed-wave Doppler images of the aortic arch^16^. However, in this OTAC model, the artifacts of the o-ring make it difficult to assess the signals. The measurement of the peak flow velocity difference between the right and left carotid artery post-procedure enables the quantification of the pressure gradient^32^. The present study could not quantify the pressure gradient because it was not measured invasively; therefore, the pressure gradient might vary among the OTAC groups, contributing to the phenotypic differences. It could be considered to measure pressure gradients noninvasively by measuring blood pressure in the right and left arms of mice to solve this limitation. If these measurements can be applied to the OTAC model, a more uniform phenotype can be achieved. Therefore, research projects on cardiac hypertrophy, fibrosis, and HFrEF will make considerable progress.

This study had several limitations. First, the echocardiographic and tissue assessments were not randomized and could be biased. However, since OTAC mice in this study showed overt hypertrophy, the effect of this may be relatively small. Second, the OTAC is performed by a single operator and the inter-operator variability has not been verified. However, in previous reports there was little inter-operator variability when o-rings were used for the pressure overload model in mice^18^. In the future, it will be necessary to verify the accuracy of the operators to ensure that inter-operator variability does not cause any significant differences in the procedure. Finally, there may be a potential bias due to the small number of mice that underwent surgery. OTAC0.45 mice scheduled for sampling at eight weeks had more cases of heart failure death from late-phase post-procedure to one week after OTAC than OTAC0.45 mice scheduled for sampling at four weeks after OTAC (5/14 vs. 0/14). Therefore, due to censoring, the Kaplan–Meier curve produced a higher survival rate than the actual sampling group at eight weeks after OTAC. Furthermore, mice that underwent OTAC0.45 had a higher mortality rate than those who underwent OTAC0.50. Therefore, the exact phenotype of OATC0.45 could not be reflected because the survived mice probably had a lower pressure overload than the dead mice. Therefore, although the degree of hypertrophy with OTAC 0.45 was expected to be higher than that with OTAC 0.50, IVSd and PWTd by echocardiography, cardiomyocyte hypertrophy by histology, and actual left ventricular weight were similar between the two groups. These results were suspected to occur due to the bias, and the difference between the two groups would resolve if the number of cases increases.

In the present study, we demonstrated a murine model of heart failure, which is a simple and highly efficient LV pressure overload model. This model will efficiently help scientists conduct research related to HFrEF and cardiac hypertrophy.

## Methods

### Experimental animals

Male, 9–10 weeks old (26.0 ± 0.5 g) C57BL/6J mice (Clea Japan, Inc., Tokyo, Japan) were randomized for sham operation (Sham) or two OTAC operation groups using 0.50 mm inner diameter o-ring (OTAC0.50) or 0.45 mm inner diameter o-ring (OTAC0.45). The o-rings for this study were confirmed with an optical microscope before the procedure, and inferior ones, such as roughness on the inner surface, were excluded. The mice were observed up to 4 and 8 weeks after the procedure. Regarding our preliminary experiment, the number of mice that underwent the OTAC procedure was set as 14 (total n = 84) in each of the six groups (Sham, OTAC0.50, or OTAC0.45 followed up for 4 or 8-weeks) at the early phase post-procedure (48 h after the procedure). All mice were maintained in ventilated cages with free access to standard rodent feed and sterile water in a temperature-controlled environment under 12/12 h light/dark cycles.

### OTAC for induction of pressure overload in mice

Mice were initially anesthetized using a mixture of medetomidine, midazolam, and butorphanol (0.3 mg/kg, 4.0 mg/kg, and 5.0 mg/kg, respectively) with intraperitoneal administration. The mice were breathing spontaneously without intubation. A mini-sternotomy was performed by making a 2–3 mm longitudinal cut in the proximal portion of the sternum, and the pectoralis muscles were blunt dissected. After retracting the thymus, the transverse aorta was identified by its location posterior to the thymus gland. For quality control, we carefully dissected the fat tissue that would alter the aortic diameter as much as possible. In OTAC groups, mice were operated with inner diameters of 0.50 mm o-ring purchased from SAKURA SEAL (Tokyo, Japan) or 0.45 mm o-ring purchased from Apple Rubber (Lancaster, NY, USA). The details on the preparation of the o-ring are described in Supplemental Fig. 3. An o-ring was applied around the transverse aorta (between the brachiocephalic and left common carotid artery) and tied with a triple knot (Fig. 1D–F). The details of the OTAC procedure are described in Supplemental Fig. 4. The incision was closed by suturing the layers using 6–0 silk sutures. Sham mice were subjected to the same operation without the insertion of an o-ring. The mice initially recovered over a warming pad and were observed throughout the perioperative period.

### Echocardiography

Transthoracic echocardiography (TTE) was performed at baseline and at weeks 1, 2, 3, 4, 5, 6, 7, and 8 after procedures using the VEVO 1100 Imaging System (VisualSonics, Toronto, Canada) with a transducer probe MS400 (VisualSonics, Toronto, Canada, with a frequency of 30 MHz). Fur was removed on the chest using depilatory cream (Veet, USA), ensuring that any residual cream was removed fully with water. For echocardiography in awake mice, we picked up the mouse by the nape and held it firmly in the palm of one hand in the supine position, with the tail held tightly between the last two fingers. The probe was gently placed over the fourth and sixth left ribs. M-mode echocardiography was recorded at the papillary muscle level from the parasternal short-axis view without sedation. In each of these captured images were included 10 to 20 cardiac cycles. Left ventricular internal dimension in end-diastolic dimension (LVIDd) and end-systolic dimension (LVIDs), the posterior wall thickness in diastole (PWTd), and interventricular septum thickness in diastole (IVSd) were measured over three cardiac cycles. Left ventricular fractional shortening (FS) was calculated using the formula FS = [(LVIDd − LVIDs)/(LVIDd)] × 100 from the M-mode measurements. These data were averaged from at least three cycles per loop.

### Tissue isolation

Mice were euthanized by cervical dislocation under sedation using a mixture of medetomidine, midazolam, and butorphanol (0.3 mg/kg, 4.0 mg/kg, and 5.0 mg/kg, respectively). The heart was excised, AT was removed, and the RV was separated from the left ventricle (including the septum). The individual chambers were weighed to assess cardiac hypertrophy. The LV was divided into the mid-papillary and apical regions. LV was snap-frozen in liquid nitrogen or stored in 10% formalin for histological analysis. The lungs were weighed, and the tibia length (TL) was measured for normalization. TL was also measured during the necropsy.

### Assessment and definition of HF

The wet lung weight was normalized to TL, as previously reported^13,20^. The lung weight/TL ratios in OTAC above the maximum value in the Sham group were regarded as having an HF phenotype, as previously defined^13^.

### Histochemistry

The LV base of the heart was rinsed in PBS and fixed in 10% formalin for a minimum of 24 h. Fixed hearts were embedded in paraffin and sectioned at the mid-ventricular plane at 4 µm. Sections were placed onto glass slides and stained with hematoxylin and eosin (HE) and picrosirius red according to the manufacturer’s protocol. Slides were imaged using a fluorescence digital microscope (BZ-X810; Keyence, Osaka, Japan). Quantitative assessments of the minor axis and area of cardiomyocytes were measured in 10 cardiomyocytes beside the pericardium on the lateral LV wall with HE staining using Image J software ver. 2.1.0/1.53c (NIH, Bethesda, MD, USA). Quantitative fractional collagen areas were calculated as the percentage of picrosirius red-stained area for cardiac tissue using the Keyence image measurement and analysis software (BZ-X810 Analyzer, Keyence, Osaka, Japan).

### Quantitative real-time reverse transcription-polymerase chain reaction (qRT-PCR)

qRT-PCR was carried out to validate the results from the histological analysis and performed in two steps. Total RNA was extracted using ISOGEN II reagent (Nippon Gene, Tokyo, Japan) according to the manufacturer’s protocol. The high-capacity RNA-to-cDNA kit protocol (Applied Biosystems, Foster City, CA, USA) was used to transcribe 1 µg of total RNA into first-strand cDNA. Then, this was followed by amplification using the GeneAmp PCR System 9700 (Applied Biosystems, Foster City, CA, USA). The reaction mixture was incubated at 37 °C for 60 min and at 10 °C for 4 min.

qRT-PCR analysis was performed for the quantitative assessment of gene expression levels. Specific mRNAs were quantified by SYBR Green qRT-PCR using a Fast Start Universal SYBR Green Master (Roche, Mannheim, Germany) and normalized to glyceraldehyde-3-phosphate dehydrogenase (*Gapdh*) expression level. Subsequently, 2 µL of cDNA template was amplified using 12.5 µL of Fast Start Universal SYBR Green Master (RoX) (Roche), 8 µL of nuclease-free water, and 2.5 µL of gene-specific primers (Supplementary Table 1) mix in a final volume of 25 µL. All qRT-PCR was carried out using an Applied Biosystems 7500 Real-Time PCR System (Applied Biosystems). The reaction mixture was incubated at 50 °C for 2 min, 95 °C for 10 min, followed by 40 cycles of 95 °C for 15 s and 60 °C for 1 min, and 95 °C for 15 s, 60 °C for 1 min, 95 °C for 15 s, and 60 °C for 15 s. Relative expression levels of target genes were calculated using the delta-delta CT method.

### Statistics

All data are presented as mean ± standard error of the mean. Statistical analyses were performed using GraphPad Prism ver. 9.1.1 (GraphPad Software, San Diego, CA, USA). Comparisons among groups were performed by one-way analysis of variance (ANOVA) with Tukey’s post hoc tests and repeated measures two-way ANOVA with Tukey’s post hoc test. Statistical significance was set at *P* < 0.05.

### Ethics

All experiments were approved by the Ehime University Animal Research Committee (approval 5TA53-1.16) and followed ARRIVE guidelines^33^. In addition, all methods were performed in accordance with the relevant guidelines and regulations.

## Supplementary Information

Supplementary Information.
