# Author Correction: O-ring-induced transverse aortic constriction (OTAC) is a new simple method to develop cardiac hypertrophy and heart failure in mice

**DOI:** 10.1038/s41598-025-89839-8

**Published:** 2025-04-15

**Authors:** Yasuhisa Nakao, Jun Aono, Mika Hamaguchi, Kayo Takahashi, Tomohisa Sakaue, Katsuji Inoue, Shuntaro Ikeda, Osamu Yamaguchi

**Affiliations:** 1 Department of Cardiology, Pulmonology, Nephrology and Hypertension, Ehime University Graduate School of Medicine, Shitsukawa, Toon, Ehime 791-0295 Japan; 2 Department of Cardiovascular and Thoracic Surgery, Ehime University Graduate School of Medicine, Toon, Ehime Japan; 3 Department of Cell Growth and Tumor Regulation, Proteo-Science Center, Ehime University, Toon, Ehime Japan

Correction to: *Scientific Reports* 10.1038/s41598-021-04096-9, published online 07 January 2022

This Article contains errors in Figure 5, panel (A) where both high-magnification images corresponding to OTAC 0.45 and OTAC 0.50 at 4 weeks (4w) are accidentally swapped between different samples. Additionally, the regions shown in the low-magnification images for OTAC 0.50 at 8 weeks (8w) do not correctly correspond to the high-magnification images. The correct Figure 5 and accompanying legend appears below.

**Figure 5:**
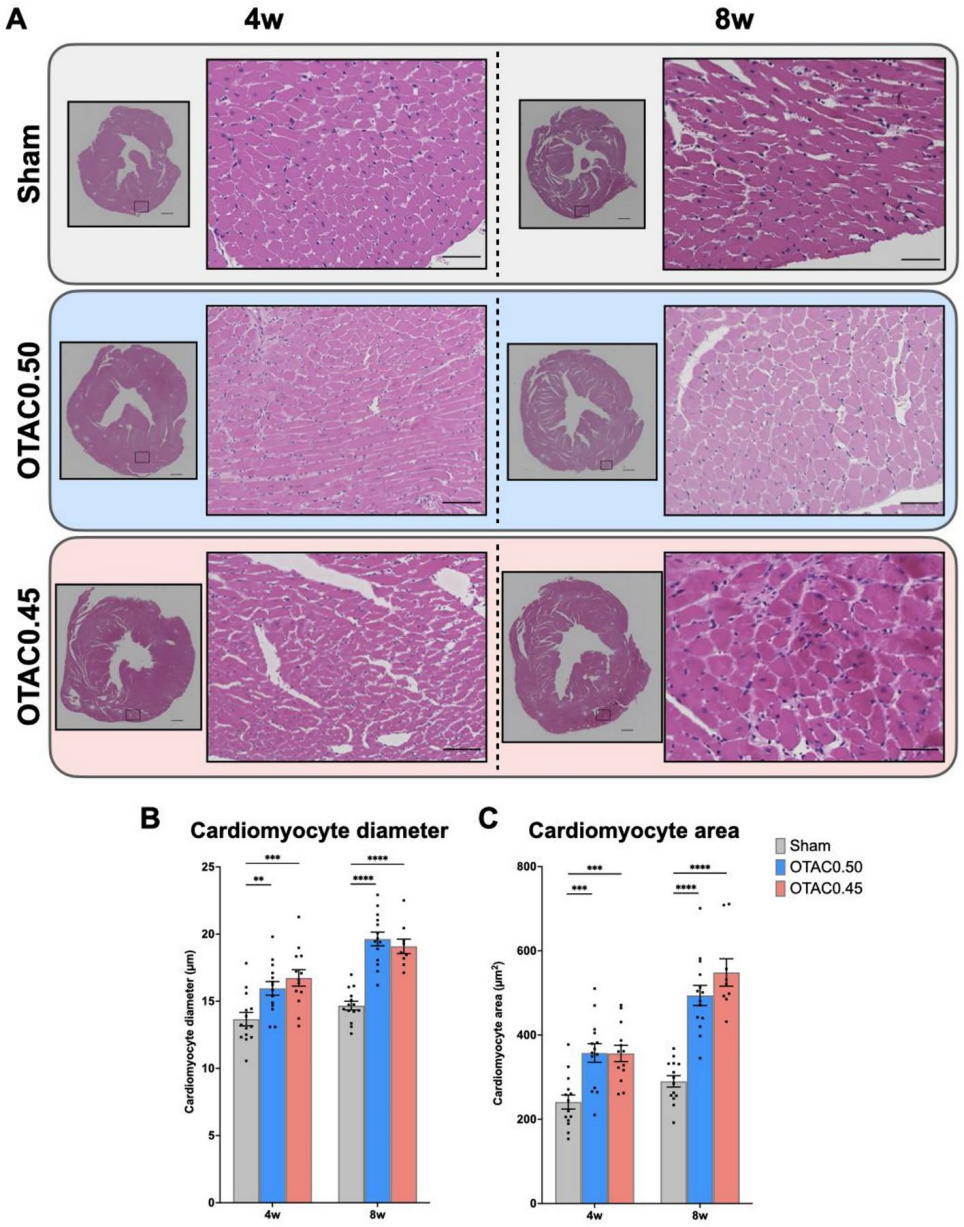
Cardiac histology at 4 and 8 weeks post-procedure. (**A**) Representative images of short-axis cardiac sections with hematoxylin and eosin staining in Sham and OTAC. Left: 4 × magnification of left ventricular at mid-ventricular sections. Scale = 500 µm. Right: 40 × magnification of a representative area. Scale = 100 µm. (**B**) Quantification of cardiomyocyte diameter of the short axis. (**C**) Quantification of cardiomyocyte area. Comparison among groups was performed by one-way ANOVA with Tukey’s post hoc tests; n = 9–14. **P < 0.01; ***P < 0.001; ****P < 0.0001.

