# Supplementary material for: O-ring-induced transverse aortic constriction (OTAC) is a new simple method to develop cardiac hypertrophy and heart failure in mice
Source: Sci Rep. 2022 Jan 7;12:85. doi: 10.1038/s41598-021-04096-9 (PMC8742050; doi:10.1038/s41598-021-04096-9)
Supplement: Supplementary file 1 — Supplementary Information. [file 41598_2021_4096_MOESM1_ESM.docx]

**Supplemental Table 1. Primer sequence for real-time PCR**

| **Gene** | **Forward** | **Reverse** |
| --- | --- | --- |
| ***Nppa*** | ATT GAC AGG ATT GGA GCC CAG AGT | TGA CAC ACC ACA AGG GCT TAG GAT |
| ***Nppb*** | CTC AAG CTG CTT TGG GCA CAA GAT | AGC CAG GAG GTC TTC CTA CAA CAA |
| ***Acta1*** | GCC AGA GTC AGA GCA GCA GAA ACT A | CAG AGC CGT TGT CAC ACA CAA GA |
| ***Col1a1*** | CCG AAC CCC AAG GAA AAG A | GTG GAC ATT AGG CGC AGG A |
| ***Col3a1*** | TCC CCT GGA ATC TGT GAA TC | TGA GTC GAA TTG GGG AGA AT |
| ***Gapdh*** | AGG TCG GTG TGA ACG GAT TTG | TGT AGA CCA TGT AGT TGA GGT CA |

**Supplemental Figure 1.**


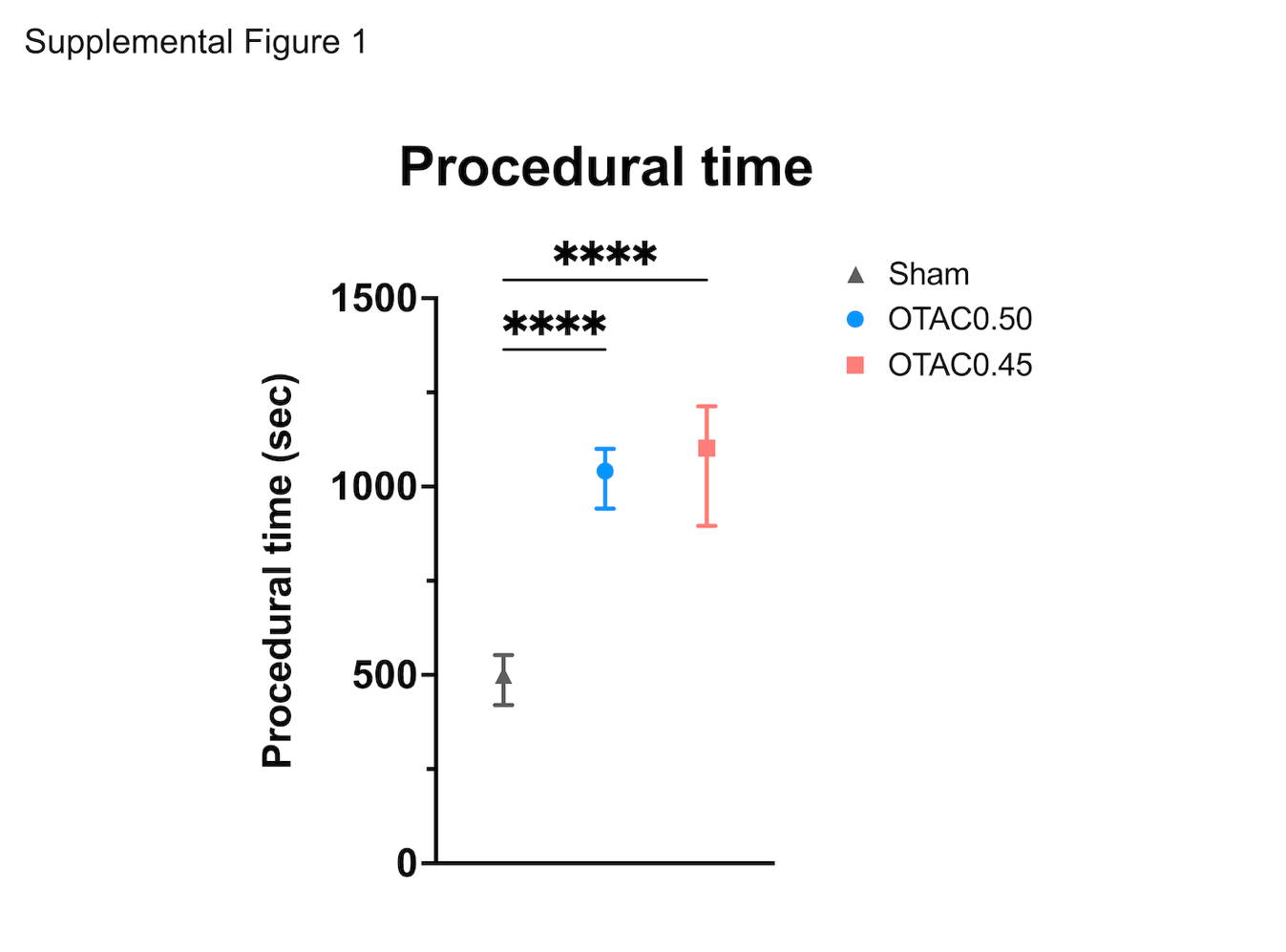


**Figure legend**

**Supplemental Figure 1:**

The average operation time (from thoracotomy to chest closure) for O-ring-induced transverse aortic constriction (OTAC) and sham procedures. The data are expressed as the mean ± standard error of the mean. O-rings had inner diameters of 0.50 mm (OTAC0.50) and 0.45 mm (OTAC0.45). Comparisons among groups were performed by ANOVA with Tukey’s post hoc test; n = 22–28. *****P*<0.0001.

**Supplemental Figure 2.**

**Supplemental Figure2:**

Echocardiographic analyses before and after OTAC, TAC, and Sham. Echocardiographic measurements of the interventricular septum in diastole: IVSd, left ventricular posterior wall thickness in diastole: PWTd, LV internal dimension in end-diastolic dimension: LVIDd, end-systolic dimension: LVIDs, and LV fractional shortening: LVFS at 0, 1, 4, and 8 weeks post-procedure.

**Supplemental Figure 3:**

**Figure legend**

**Supplemental Figure 3:** Preparing for the O-ring.

**1**: Tools to be used. **A**: Two short pieces of 6-0 silk thread. **B**: An o-ring. **C**: Scissors. **D**: Tweezers (can be curved or straight tip). **E**: A 29G needle (can be done without a syringe). **2-3**: Penetrate the 29G needle vertically through the o-ring. **4**. Pass one thread from the tip of the needle to the back (yellow arrow). **5**: The needle is pulled out (yellow arrow), and the thread remains. **6**: Finish the process for one thread. **7**: The other thread is passed through the needle in the same way, near the first thread that was passed through. **8**: The thread is passed through the needle to the end (yellow arrow). **9**: The needle is pulled out (yellow arrow), and the thread remains. **10**: The state after two threads are passed through the o-ring. **11**: Cut the o-ring using scissors between the two threads. **12**: Confirm that the o-ring is cut. **13**: The o-ring with threads is ready for OTAC. **14:** The clip art of an o-ring with threads.

**Supplemental Figure 4:**

**Figure legend**

**Supplemental Figure 4:** The process of OTAC using a model.

**1**: Tools to be used. **A**: An o-ring with the threads. **B**: Scissors. **C**: Tweezers (curved and blunt tip). **D**: Tweezers (curved and sharp tip). **E**: A ligation device with a hole in the tip. **2**: Pass one side of the o-ring thread through the hole in the tip of the device. **3**: Pass the ligation device under the transverse aorta from the cranial to the caudal side (yellow allow). Rt: The right side of the mouse. Lt: The left side of the mouse. **4-5**: Pull the thread out of the device from the caudal side. **6-7**: Pull the thread (yellow allow) and pass the O-ring under the transverse aorta from the cranial to the caudal side. **8-9**: Pull both threads from the cranial and the caudal sides, and clamp the O-ring into the transverse aorta. **10**: Tie the threads with triple knots on the right side of the mouse. **11**: Tie the threads with triple knots on the left side of the mouse. **12**: The end of OTAC.
